# Supplementary material for: Real-World Data Validation of NAPOLI-1 Nomogram for the Prediction of Overall Survival in Metastatic Pancreatic Cancer
Source: Cancers (Basel). 2023 Feb 5;15(4):1008. doi: 10.3390/cancers15041008 (PMC9954707; doi:10.3390/cancers15041008)
Supplement: Supplementary file 1 [file cancers-15-01008-s001.zip › Supplementary Figures.pdf]

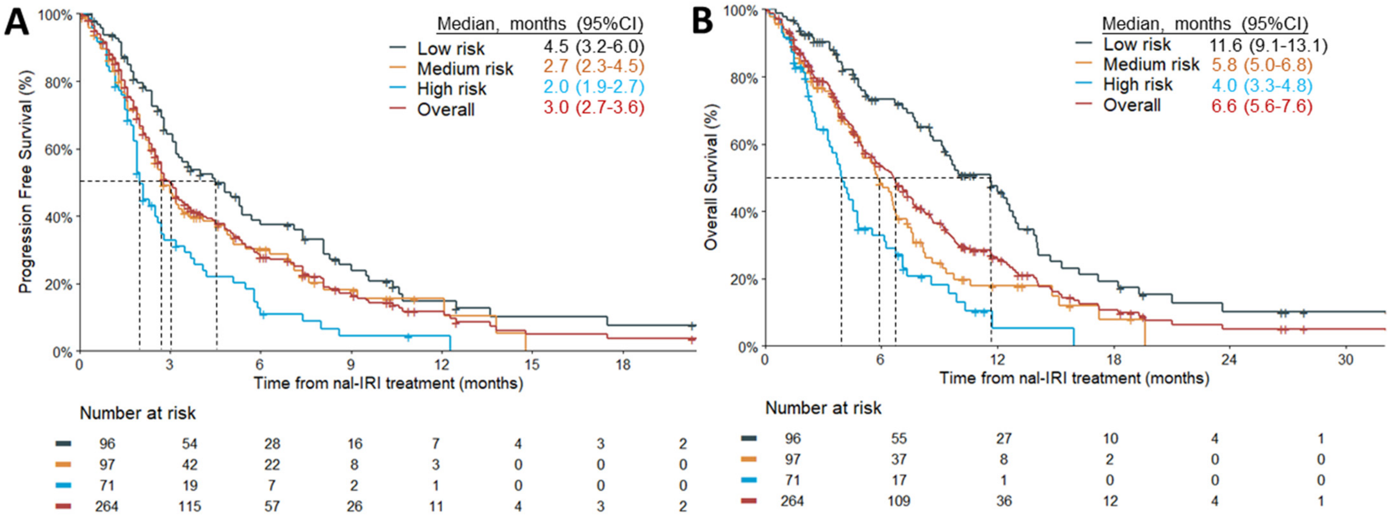

Supplementary Figure S1. Sensitivity analysis of survival in patients without missing value in NAPOLI-1 nomogram (n=264). Progression free survival (A) and overall survival (B) of entire and different risk groups.

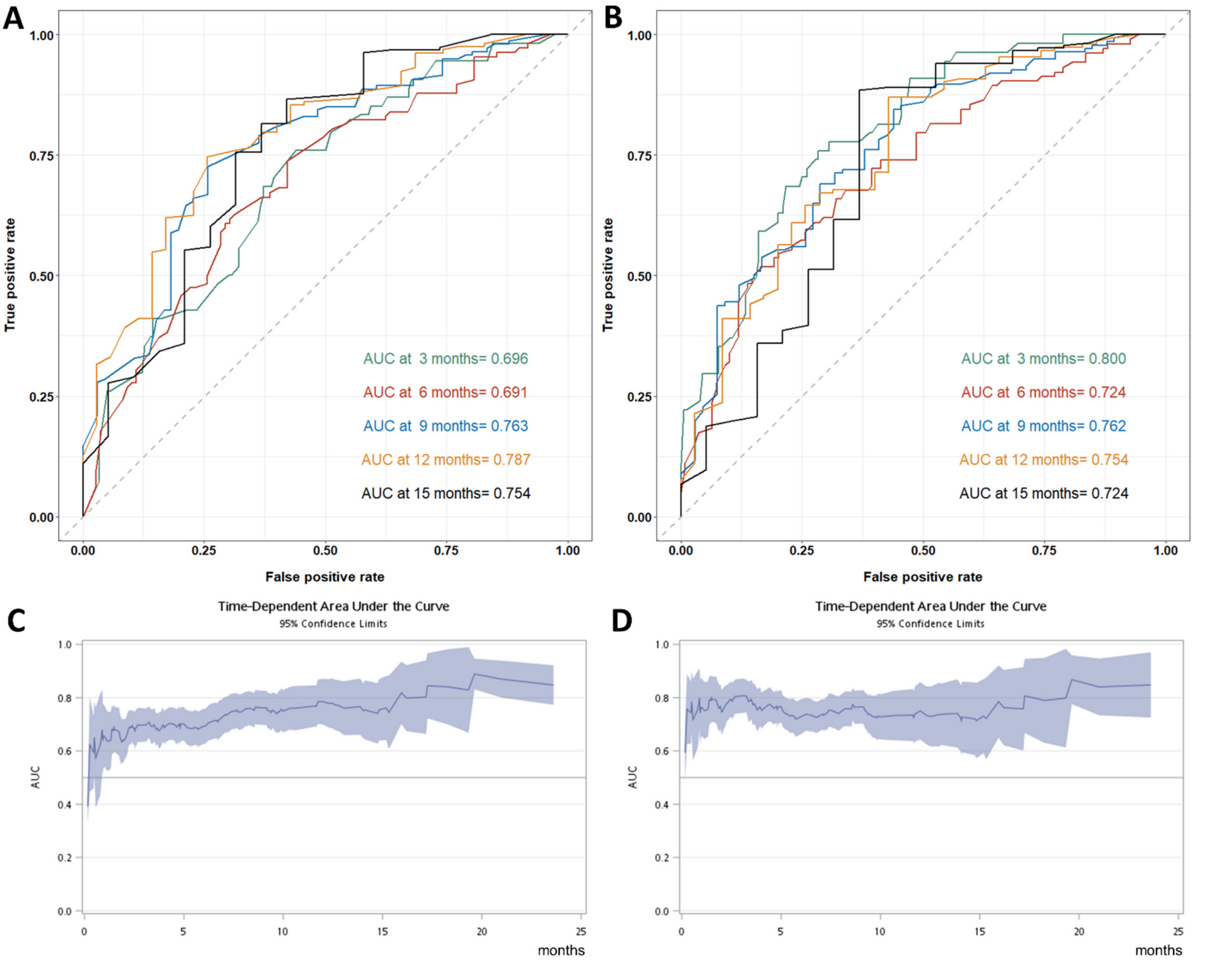

Supplementary Figure S2. Sensitivity analysis of model performance in patients without missing value in NAPOLI-1 nomogram (n=264). Area under the receiver operating characteristic curve (AUC) at 3,6,9,12 and 15 months in NAPOLI-1 nomogram (A) and NAPOLI-1 nomogram with cumulative dose at 6 weeks (B).

Time-dependent AUC of NAPOLI-1 nomogram(C) and NAPOLI-1 nomogram with cumulative dose at 6 weeks (D).

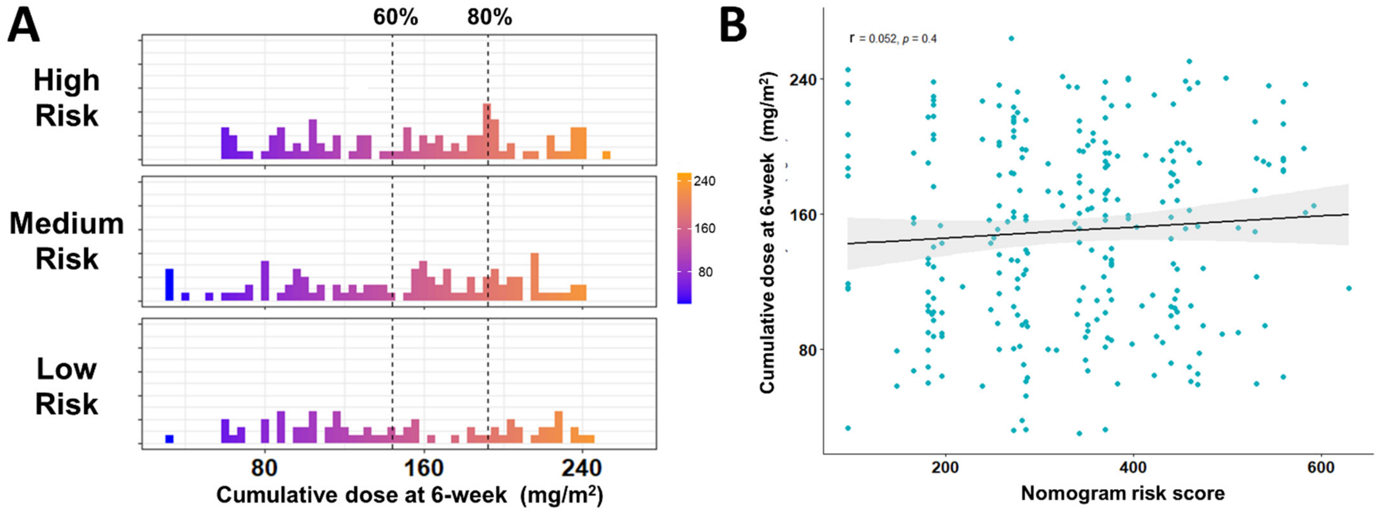

Supplementary Figure S3. Sensitivity analysis of dose distribution in patients without missing value in NAPOLI-1 nomogram (n=264). (A) Distribution of cumulative dose at 6-week in patients without missing value in different risk groups (n=264). **(B)** Spearman correlation of nomogram risk score and cumulative dose at 6-week in patients without missing value (n=264).

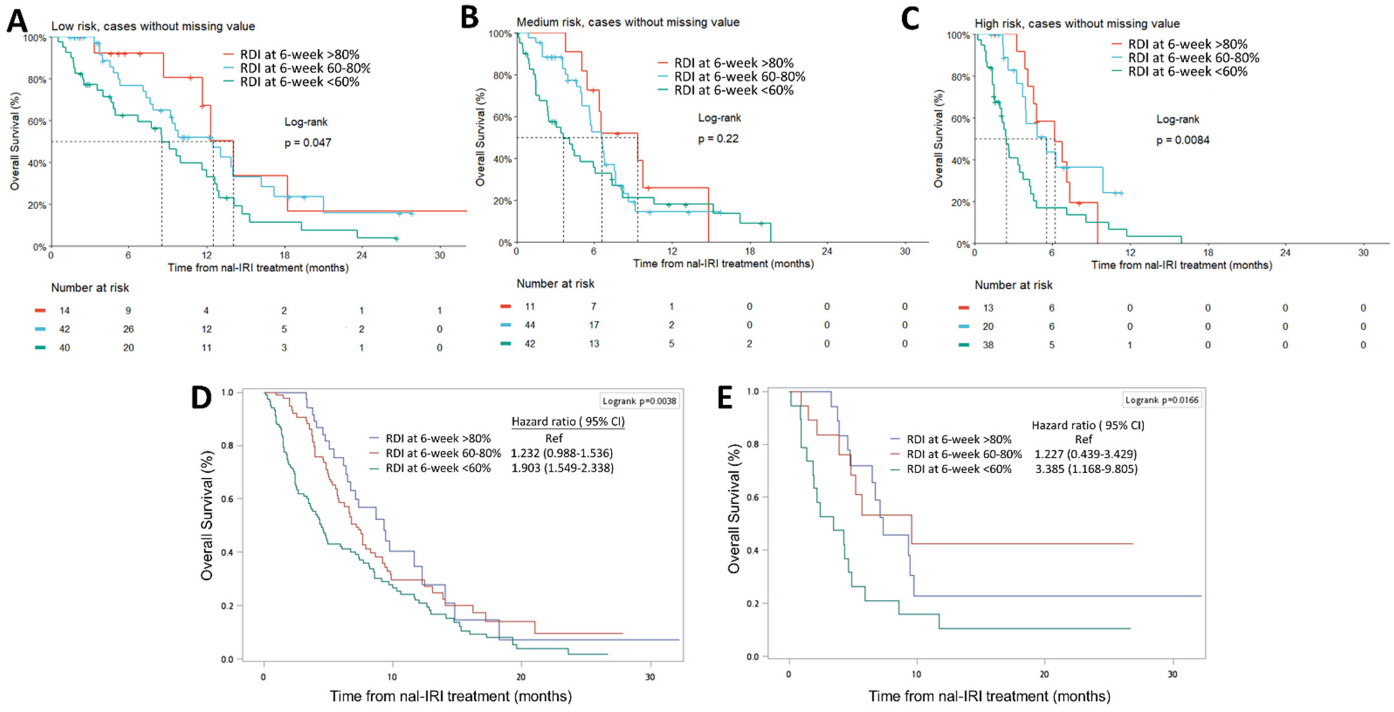

Supplementary Figure S4. Sensitivity analysis of survival in patients without missing value in NAPOLI-1 nomogram (n=264). Overall survival in patients without missing value in low risk groups (A), medium risk groups (B) and high risk groups (C). (D) Overall survival weighted by inverse probability of weights (IPW) in different cumulative dose groups without missing value. (E) Overall survival after 1:1:1 three group matching by NAPOLI-1 nomogram risk score in different cumulative dose groups without missing value (n=19 in each group).
