# Supplementary material for: Real-World Data Validation of NAPOLI-1 Nomogram for the Prediction of Overall Survival in Metastatic Pancreatic Cancer
Source: Cancers (Basel). 2023 Feb 5;15(4):1008. doi: 10.3390/cancers15041008 (PMC9954707; doi:10.3390/cancers15041008)
Supplement: Supplementary file 1 [file cancers-15-01008-s001.zip › Supplementary Tables.pdf]

Table S1. Multivariable Cox regression of factors in NAPOLI-1 nomogram (n = 473)

	HR (95% CI)	Wald p-Value
Baseline ECOG Performance Score, ≥1 vs. 0	0.840 (0.642-1.100)	0.2063
Baseline albumin, ≥4 g/dL vs. <4 g/dL	0.721 (0.568-0.915)	0.0073
Neutrophil lymphocyte ratio, ≤5 vs. >5	0.650 (0.511-0.827)	0.0004
No liver metastasis vs. liver metastases	0.653 (0.509-0.839)	0.0008
Baseline CA19-9 ≤ 1542 vs. >1542	0.574 (0.457-0.721)	<0.0001
Stage I to III vs. stage IV at diagnosis	0.763 (0.594-0.979)	0.0338
Body mass index >25 vs. ≤25	1.035 (0.764-1.403)	0.8201

Table S2. Multivariable Cox regression of other covariates with NAPOLI-1 nomogram risk score (n =473)

	HR (95% CI)	Wald p-Value
NAPOLI-1 nomogram risk score	0.996 (0.995-0.997)	<0.0001
Age>65 vs. ≤65	1.255 (0.989-1.593)	0.0612
Gender, male vs. female	1.273 (1.009-1.607)	0.0412
Relative dose intensity at 6-week	0.991 (0.989-0.994)	<0.0001
Primary tumor location: head of pancreas vs. other	0.912 (0.712-1.167)	0.4643
Whipple operation: yes vs. no	0.697 (0.489-0.996)	0.0476
Prior exposure of fluorouracil, yes vs. no	1.506 (1.084-2.092)	0.0145
Prior exposure of irinotecan, yes vs. no	1.259 (0.900-1.762)	0.1776
Prior exposure of platinum, yes vs. no	1.253 (0.965-1.626)	0.0898
Number of metastatic sites	1.075 (0.938-1.233)	0.2949

Table S3. Model in patients without missing value (n=264)

Model 1<br>NAPOLI-1 nomogram			Model 2<br>NAPOLI-1 nomogram and cumulative dose		
**Parameter**	**HR (95%CI)**	**P-value**	**Parameter**	**HR (95%CI)**	**P-value**
Nomogram risk score	0.996 (0.995-0.997)	<0.0001	Nomogram risk score	0.996 (0.995-0.997)	<0.0001
			Relative dose intensity at 6-week >80%	Reference	-
			Relative dose intensity at 6-week 60-80%	1.310 (0.813- 2.110)	0.2658
			Relative dose intensity at 6-week <60%	2.092 (1.332- 3.287)	0.0013
